# An fMRI study on cortical responses during active self-touch and passive touch from others

**DOI:** 10.3389/fnbeh.2012.00051

**Published:** 2012-08-07

**Authors:** Rochelle Ackerley, Eusra Hassan, Andrew Curran, Johan Wessberg, Håkan Olausson, Francis McGlone

**Affiliations:** ^1^Department of Physiology, University of GothenburgGothenburg, Sweden; ^2^Magnetic Resonance and Image Analysis Research Centre, University of LiverpoolLiverpool, UK; ^3^Alder Hey Children's NHS Foundation TrustLiverpool, UK; ^4^School of Natural Sciences and Psychology, Liverpool John Moores UniversityLiverpool, UK

**Keywords:** glabrous, hairy, motor, sensorimotor, skin, somatosensory, stroking

## Abstract

Active, self-touch and the passive touch from an external source engage comparable afferent mechanoreceptors on the touched skin site. However, touch directed to glabrous skin compared to hairy skin will activate different types of afferent mechanoreceptors. Despite perceptual similarities between touch to different body sites, it is likely that the touch information is processed differently. In the present study, we used functional magnetic resonance imaging (fMRI) to elucidate the cortical differences in the neural signal of touch representations during active, self-touch and passive touch from another, to both glabrous (palm) and hairy (arm) skin, where a soft brush was used as the stimulus. There were two active touch conditions, where the participant used the brush in their right hand to stroke either their left palm or arm. There were two similar passive, touch conditions where the experimenter used an identical brush to stroke the same palm and arm areas on the participant. Touch on the left palm elicited a large, significant, positive blood-oxygenation level dependence (BOLD) signal in right sensorimotor areas. Less extensive activity was found for touch to the arm. Separate somatotopical palm and arm representations were found in Brodmann area (BA) 3 of the right primary somatosensory cortex (SI) and in both these areas, active stroking gave significantly higher signals than passive stroking. Active, self-touch elicited a positive BOLD signal in a network of sensorimotor cortical areas in the left hemisphere, compared to the resting baseline. In contrast, during passive touch, a significant negative BOLD signal was found in the left SI. Thus, each of the four conditions had a unique cortical signature despite similarities in afferent signaling or evoked perception. It is hypothesized that attentional mechanisms play a role in the modulation of the touch signal in the right SI, accounting for the differences found between active and passive touch.

## Introduction

The brain receives afferent information from the activation of mechanoreceptors in the skin during interactions with the environment. The present study focuses on the cortical representations from active, self-touch and passive (other) touch to the palm and arm. There are differences in the types of low-threshold mechanoreceptors found in the glabrous skin of the palm compared to the hairy skin on the arm (for an overview, see Macefield, 2005). The mechanoreceptors on the glabrous skin allow high discriminatory abilities for touch, whereas the input from hairy skin does not give such discrimination. Despite these differences, glabrous and hairy skin are both sensitive to touch; a recent study has shown that psychophysical ratings of the intensity and the pleasantness of touch were not different between the skin of the palm and the arm (McGlone et al., 2012). However, the study also found that using a Touch Perception Task (from Guest et al., 2011), subjects used more sensory descriptors when evaluating touch to the palm, whereas they used more emotional descriptors for touch to the arm, indicating that touch is processed over many cognitive levels.

The high discriminatory ability from human glabrous skin (e.g., the ventral surfaces of the hands and feet) is based on inputs from four main classes of low-threshold mechanoreceptors, namely: rapidly-adapting types I (RAI; Meissner's corpuscles) and II (RAII; Pacinian corpuscles), and slowly-adapting types I (SAI; Merkel's disks) and II (SAII; Ruffini's endings). These afferents are in the Aβ conduction range of myelinated mechanoreceptors and send information to the brain very quickly (conducting at 36–73 m/s; Kakuda, 1992) at a high temporal resolution (Perge et al., 2012). This type of mechanoreceptive input provides an excellent source of information during discriminative touch; the incoming, high-quality tactile information can be compared in areas of the brain such as SI. This capacity can be demonstrated in ways such as using two-point discrimination tests, where the fingertip skin has high discriminatory ability (2–4 mm; Bickley and Szilagyi, 2007).

Hairy skin is defined as the non-glabrous and non-mucocutaneous skin that covers the majority of the body surface. Hairy skin does not contain RAI mechanoafferents but instead, includes hair, field and C-tactile (CT) afferents. These afferents have myelinated axons (e.g., Aβ terminal hair and field units; Vallbo et al., 1995), or are unmyelinated (CT; Vallbo et al., 1993). The density of myelinated afferents in hairy skin is much less than in glabrous skin (Provitera et al., 2007). In contrast to glabrous skin, hairy skin has a much lower discriminatory ability (e.g., 30–40 mm in the two-point discrimination test; Bickley and Szilagyi, 2007), but is nevertheless sensitive to touch; in fact, CT afferents respond to <250 mg force (Vallbo et al., 1993; Wessberg et al., 2003; Cole et al., 2006). The touch information relayed to the brain from CT afferents is comparatively lower in temporal resolution due to the slower conduction velocity and more variable firing discharge (Vallbo et al., 1993, 1999; Wessberg et al., 2003).

Touch information is described classically as having a somatotopical representation in the contralateral SI (Penfield and Rasmussen, 1950), where discriminative and integrative aspects of touch are processed, such as form, texture, shape, and size (Hsiao, 2008). Tactile input is also processed in other cortical areas and the information flow can be split into dorsal and ventral streams (Romo et al., 2002). The dorsal stream sends information to Brodmann areas (BA) 5 and 7 and has been associated with processing during active touch, such as during voluntary movements (Shanks et al., 1985; Cavada and Goldman-Rakic, 1989; Romo et al., 2002). The ventral stream has been more associated with discrimination, feature and pattern recognition, and flows though lateral somatosensory areas, such as the secondary somatosensory cortex (SII), but also includes activation of premotor and prefrontal areas (Pons et al., 1987; Carmichael and Price, 1995; Romo et al., 2002). Both of these streams include reciprocal connections with motor areas and there are within- and between-hemispheric somatosensory connections (Goldring et al., 1970; Fabri et al., 1999; Tommerdahl et al., 2006; Eickhoff et al., 2008; Ragert et al., 2011; Schäfer et al., 2012). In active touch, the motor system must communicate with the somatosensory system during behavior, such as in the fine-detail exploration of a surface. Here, there must be an exchange of sensory and motor information and the primary motor cortex (MI) also receives direct input from SI (Huerta and Pons, 1990). It is likely that combinations of these areas work together to integrate and process touch information and shape how we act on it.

The present study aims to investigate the differences between the cortical representations from active and passive forms of touch. The mode of touch relates to the skin site: active touch is typically carried out by the hands whereas the rest of the body is more involved in passive touch, for example, from another person. Active, self-touch and the passive touch from another will engage similar afferent mechanoreceptors on the same skin site but may be processed differently. Active touch is self-governed, where there is a motivation and an expectation of upcoming sensory input. A feed-forward efference copy signal predicting the expected outcome of movements is sent to somatosensory areas, such as SI and the cerebellum, for movement-related gating of the incoming sensory input (Chapin and Woodward, 1981; Blakemore et al., 1998). This has been hypothesized to be for sensory cancellation e.g., how you cannot tickle yourself because an internal forward model captures the relationship between the motor efference copy and the predicted sensory consequences of the action (Weiskrantz et al., 1971; Blakemore et al., 1998, 2000). Sensory cancellation allows you to attend to any unexpected parts of the input, while ignoring the expected sensory feedback. Conversely, during passive touch, an expectation of touch may be present, but there is no motor efference copy to nullify or cancel the subsequent, sensory input.

Previous neuroimaging studies have found that active touch produces sensations that are less intense, compared to that from passive touch, in part due to sensory cancellation (Blakemore et al., 1998, 1999). In these studies, the BOLD signal in SI contralateral to the touched surface was found to be significantly lower for active touch. It is reasoned that incoming signals are attended to less if they match the expected parameters, so can be ignored, thus a decrease in the signal is observed. The finding of decreased activity in SI for active compared to passive touch is controversial. Some studies have found a decreased SI signal, for example, in animal *in vivo* recordings (Chapin and Woodward, 1981; Jiang et al., 1991), electroencephalography (EEG; Abbruzzese et al., 1981; Tapia et al., 1987), magnetoencephalography (Hesse et al., 2010), and fMRI (Blakemore et al., 1998). However, other animal *in vivo* electrophysiological experiments (Chapman and Ageranioti-Bélanger, 1991; Ageranioti-Bélanger and Chapman, 1992) and a recent fMRI experiment (Simões-Franklin et al., 2011) have found different results: an increased contralateral SI signal during active touch.

Information gained from active touch and dynamic passive touch has been shown to be perceptually similar (Lederman, 1981; Verrillo et al., 1999). It is therefore likely that the motor component in active touch can be countermanded to the extent that movement-related gating has virtually no effect. Similar effects have been seen in other sensory-motor tasks, such as the interaction of vision with head movement, where a cancellation signal occurs but has little effect on the response (Ackerley and Barnes, 2011). It is to be expected that all available sensory and motor information is used together in processing the information from touch. The present study uses fMRI to detect significant modulations in the BOLD signal to active (self-touch) and passive stroking (touch via the experimenter) of the glabrous skin (palm) and hairy skin (arm), using a soft, cosmetic brush for stimulation. This was specifically used to activate low-threshold mechanoreceptors and investigate the cortical differences within and between the touch conditions. Therefore, despite similarities in perception, we hypothesize that each condition (active, passive, hairy, or glabrous) has a unique cortical representation.

## Materials and methods

The study was carried out using a Siemens 3T MRI scanner with an eight channel head coil, at the University of Liverpool, UK. All participants were screened prior to taking part for safety and were supplied with information about the study, which conformed to local ethical approval and was performed in accordance with the Declaration of Helsinki. Written, informed consent was obtained for a total of 12 healthy, male volunteers (aged 18–35). The study compared intra-personal touch where the person actively stroked themselves (self-touch) using a soft cosmetic brush (width = 4 cm), with inter-personal touch where the participant was stroked using an identical brush, by the experimenter (other-touch). The body sites stroked were the palm (glabrous skin in the middle of the left palm) and the arm (dorsal hairy surface of the left mid-arm). Each stroked area was approximately 10 cm long and the stroking velocity was between 6 and 8 cm/s. The participant could not see the experimenter during the session, although, they were instructed about the type of stroking via a viewing screen.

The paradigm consisted of four different randomized touch conditions that were repeated eight times each. Each block consisted of 9 s of stroking, then 6 s of rest. During the stroking, the participant saw a continuous visual instruction (e.g., “Stroke your palm” and “Your arm will be stroked”), which was projected onto a screen in front of the participant, viewed via a mirror on the head coil. The participants were made aware of the timings and task beforehand, and they practiced the conditions before going into the scanner, and also in the scanner before the experiment started. This aided in gaining consistency in stroking within and between participants and also allowed for similar forces to be applied (~0.8 N). To minimize motion artifacts that would produce head movement in the scanner, the participants were instructed to make only small arm movements during brushing.

The parameters used for the functional gradient echo-planar imaging sequence were: in-plane resolution = 64 × 64 matrix, field of view = 192 mm, flip angle = 90°, TR = 3000 ms, TE = 30 ms, and inter-slice interval = 71 ms over 160 volumes (42 slices for whole-brain coverage at a resolution of 3 × 3 × 2.5 mm with slice gap = 0.5 mm). A T_1_-weighted, high resolution anatomical scan (176 slices at 224 × 256 mm coverage, 1 mm isotropic voxels) was conducted either before or after the paradigm. Brain Voyager (v2.4, Brain Innovation, Maastricht, Netherlands) was used for the analysis of the fMRI data and Statistical Package for the Social Sciences (SPSS) (IBM, Armonk, NY) was used for further statistical investigations in region-of-interest (ROI) analyses. The raw fMRI data were imported and preprocessed with the following standard parameters: slice scan time correction, 3D motion correction, spatial smoothing (with a Gaussian filter of full width half maximum = 3 mm in the space domain), and temporal filtering. All of the subjects had <3 mm motion and <3° translation. Anatomical data were imported and corrected for image intensity inhomogeneity (a step that included brain extraction from surrounding skull and tissue, and the segregation of gray and white matter), before conversion to Talairach space. The preprocessed functional data were coregistered with the original anatomical data to make a volume time course file. Data were saved in neurological convention (where left-is-left and vice versa) at a resolution of 3 × 3 × 3 mm.

The processed functional data were linked to the Talairach brain (Talairach and Tournoux, 1988) and a single-subject general linear model (GLM) was carried out with four predictors (the stroking conditions) using Z-scores as a change from the signal baseline (rest), with a hemodynamic response function applied. Each participant's data was inspected for significant BOLD activity changes from the resting baseline between the conditions. A multi-subject group-level GLM was then carried out, for random effects significant differences in the BOLD signal. The different stroking conditions were contrasted against the baseline rest period and significantly activated voxels were sought at a false discovery rate (FDR) of *q* < 0.05, which corrected for multiple comparisons (see Genovese et al. (2002), Goebel et al. (2006), and Gordon et al. (2011) for use of FDR correction to increase statistical power over Bonferroni correction). ROIs were identified based on the results of the contrasts, although, the main targets for analysis were sensory and motor cortical areas in the brain (e.g., SI, SII and MI). The Talairach co-ordinates for regions showing significant changes in the BOLD signal were entered into Talairach Client (Talairach.org; Lancaster et al., 1997, 2000) to determine the exact brain area modulated. Beta-weights (relating to the BOLD z-score amplitude) were computed for each participant in each condition in ROIs. SPSS (IBM, Armonk, NY) was used to calculate significant differences between touch conditions using analysis of variance [ANOVA; 2 × 2 design: mode of stimulation (active or passive) and the body site touched (palm or arm)], with *post-hoc* multiple comparisons where the factors were contrasted separately (sought at *p* < 0.05).

## Results

**Figure 1:**
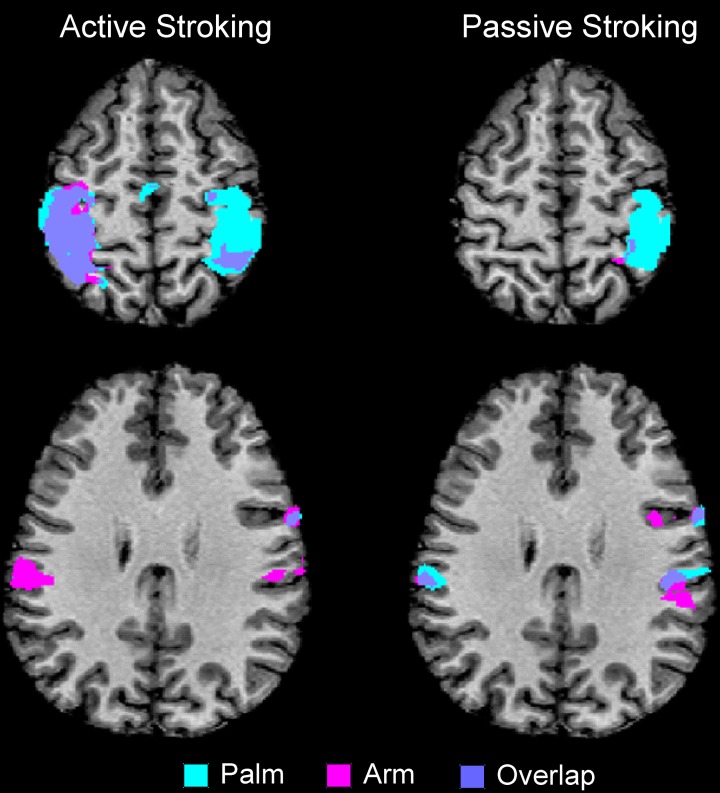
**Overview of the main regions where positive BOLD signal changes were found for active and passive stroking on the palm and arm, compared to the resting baseline**. There were clear differences between active and passive touch, as can be seen in the BOLD signal in the left SI and MI (compare the top two panels). There were also body site differences: the right SI had large regions of activity from palm stroking, whereas much less activity was found to arm stroking (see also Table 1). There was also bilateral SII activation to arm stroking, whereas bilateral activity for palm stroking was only found for passive touch (in active touch to the palm, there was only right SII activity). The maps are to neurological convention (left is left).

**Figure 2:**
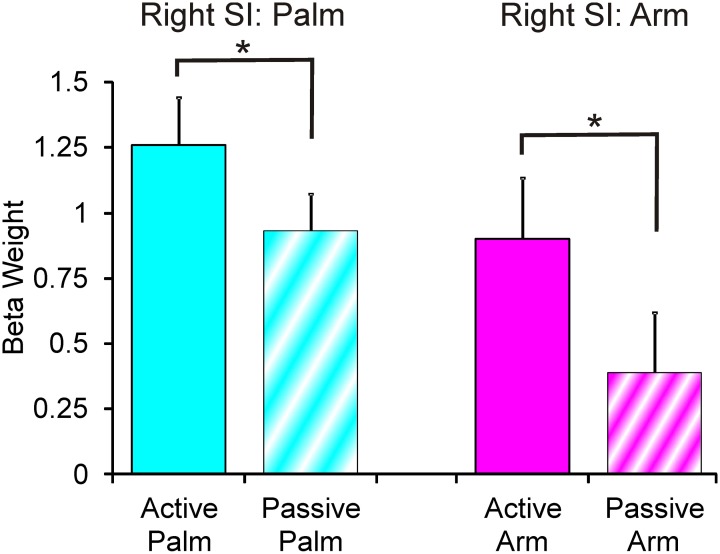
**Beta weights from the right SI palm and arm respective areas**. There were significant differences for the palm SI BA03 region and arm SI BA03 region (see Table 1 for area details), where active stroking gave significantly higher beta values than passive stroking. Error bars show ±1 standard error.

The results from comparing the different touch conditions showed striking differences in sensorimotor areas. From inspection of each participant's data, and as found previously (Olausson et al., 2002; Björnsdotter et al., 2009), the data from some participants (*n* = 4) showed less BOLD activation, due to increased head movement and also drowsiness may have played a factor. Although, these participants' data followed similar trends, they were not used in calculations. Overall in the data, there were more extensive areas showing positive BOLD signal changes for palm compared to arm stroking in the right sensorimotor cortex, irrespective of whether the stroking was active or passive (Figure 1). A somatotopical representation was found in the right SI (BA03): the palm representation was in the middle of the post-central gyrus and covered a large area (see Figure 1), whereas the arm representation was further lateral (not shown in Figure 1 due to the slice orientation; see Table 1 for details). In both of these specific body site regions in BA03, active touch gave significantly higher beta values than passive touch to the same area, respectively (*p* < 0.05; Figure 2). Other areas in the left sensorimotor cortex showed significant positive BOLD modulations compared to the resting baseline for touch to the palm and arm (see Table 1), however, none of these regions showed significant differences between the active and passive touch beta values.

**Table 1:** **Overview of all the cortical regions showing significant differences from the resting baseline for each touch conditions**.

	***Brodmann area***	**Peak Talairach co-ordinates**			**Maximum *t*-score**	**Number of voxels**
		***x***	***y***	***z***
**ACTIVE PALM VS. REST**						
Left premotor cortex	6	−4	−15	53	8.96	203
	6	−58	2	39	9.56	710
Left precentral gyrus (MI)	4	−29	−23	67	11.93	912
Left postcentral gyrus (SI)	3	−35	−30	57	12.66	956
	2	−54	−28	50	11.49	491
	2	−60	−22	35	9.70	918
	40	−40	−44	53	12.80	497
Left somatosensory association cortex	5	−34	−40	59	12.46	964
	7	−21	−54	61	4.81	483
	7	−35	−54	54	7.02	650
Right premotor cortex	6	7	−19	72	5.42	586
	6	57	1	39	7.16	601
Right precentral gyrus (MI)	4	31	−20	63	13.28	792
Right postcentral gyrus (SI)	3	37	−31	58	13.15	955
	40	35	−44	52	14.31	845
	2	56	−22	41	10.04	900
Right operculum (SII)	40	58	−18	24	7.61	523
Right insula	13	49	−18	17	3.30	151
Right inferior frontal gyrus	44	54	7	14	3.67	87
**ACTIVE ARM VS. REST**						
Left premotor cortex	6	−4	−12	54	7.11	708
	6	−58	0	39	6.57	324
Left precentral gyrus (MI)	4	−30	−21	66	11.66	992
Left postcentral gyrus (SI)	3	−35	−29	59	11.66	1000
	2	−52	−26	50	9.41	939
	2	−59	−22	36	11.09	989
	40	−36	−42	53	9.67	998
Left somatosensory association cortex	5	−34	−38	59	10.75	945
	7	−26	−65	53	3.14	148
	7	−35	−54	54	6.57	380
Left operculum (SII)	40	−59	−23	23	6.11	452
Right premotor cortex	6	7	−10	62	5.47	47
	6	58	0	37	8.72	503
Right postcentral gyrus (SI)	3	57	−21	41	9.63	755
	40	36	−42	54	7.83	438
Right operculum (SII)	40	53	−22	27	5.28	127
Right inferior frontal gyrus	44	54	4	14	6.70	191
**PASSIVE PALM VS. REST**						
Left premotor cortex	6	60	0	35	6.42	394
Left postcentral gyrus (SI)	2	−60	−23	34	8.93	939
*Left postcentral gyrus (SI)*	*3*	−*36*	−*31*	*59*	−*11.87*	*632*
Left operculum (SII)	40	−59	−23	24	6.24	616
Left insula	13	−49	−38	23	3.41	142
	13	−40	−5	11	4.10	150
Right precentral gyrus (MI)	4	40	−18	58	9.57	900
Right postcentral gyrus (SI)	3	40	−31	59	8.66	984
	2	56	−22	35	7.98	772
	40	36	−40	56	8.72	991
Right operculum (SII)	40	52	−22	21	6.03	784
	40	57	−31	24	4.91	553
Right insula	13	43	−19	20	5.07	450
	13	40	−14	11	4.75	217
Right inferior frontal gyrus	44	54	3	17	3.37	71
**PASSIVE ARM VS. REST**						
*Left postcentral gyrus (SI)*	*3*	−*35*	−*31*	*59*	−*12.98*	*570*
Left operculum (SII)	40	−61	−24	32	5.78	485
Left insula	13	−50	−37	23	3.87	73
Right postcentral gyrus (SI)	3	58	−21	37	5.91	346
	40	27	−43	56	3.64	17
Right operculum (SII)	40	52	−22	21	4.73	637
	40	58	−34	26	6.59	840
Right insula	13	42	−31	21	5.54	557
	13	39	−15	12	3.64	89
Right inferior frontal gyrus	44	54	1	17	4.18	211
Right superior temporal gyrus	22	67	−37	16	4.04	217
Right angular gyrus	39	47	−55	8	3.88	189

The table details the Brodmann area, Talairach co-ordinates (x, y, z) of the peak of the BOLD signal, the maximum t-score and the number of voxels in each region. The italicized numbers denote a significant negative BOLD signal; all other regions show significant positive BOLD signals compared to the baseline.

Comparing touch to the palm and arm, there were other region-specific differences. For touch to the arm, there were significant BOLD signals found bilaterally in SII during both active and passive touch, compared to the resting baseline. For touch to the palm, there was a bilateral activation of SII during passive touch compared to rest, however, during active stroking of the palm there was only significant BOLD signal changes in the right SII (see Figure 1 and Table 1). During touch to the palm, the right MI showed significant positive BOLD signals compared to the resting baseline. There were also significant positive BOLD signals in the right premotor cortex during active touch to the palm and arm; however, there were no right-sided motor area activations for passive stroking of the arm (see Table 1).

**Figure 3:**
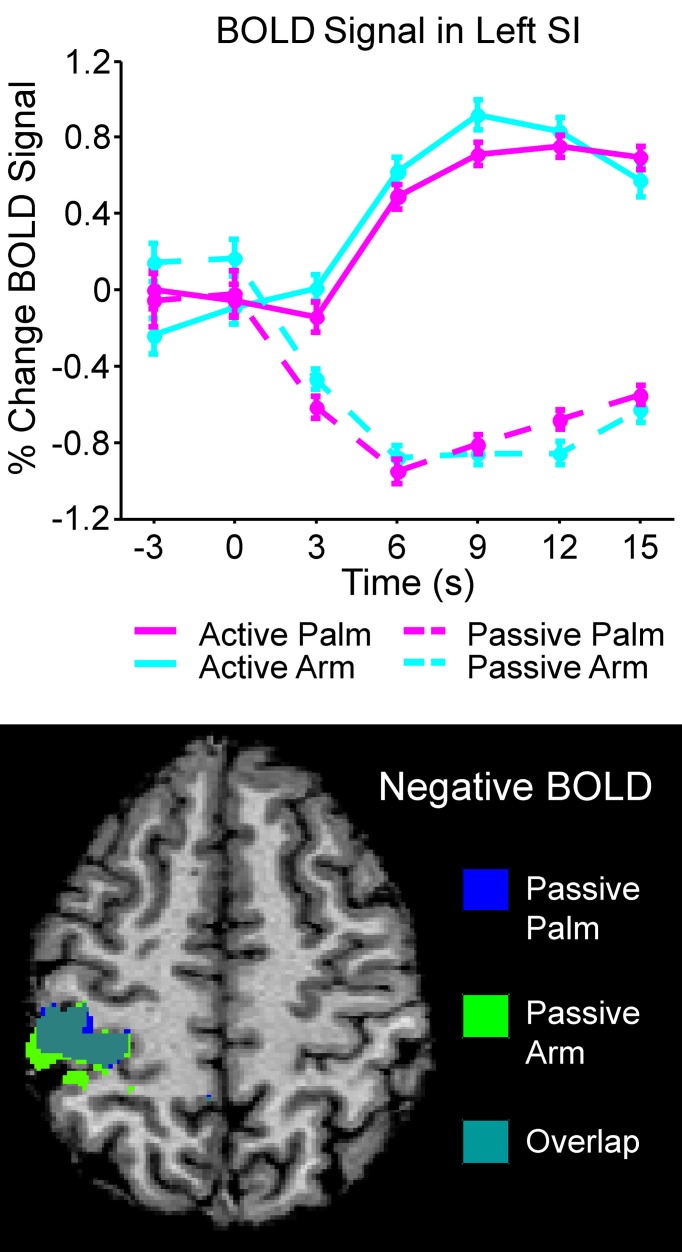
**Overview of the left SI area that showed significant negative BOLD during passive stroking**. The top graph shows the time courses of all the conditions in the left SI BA03 area (see Table 1 for more details); in active touch, there was a significant positive BOLD signal changes, whereas in passive touch, there was a significant negative BOLD signal. The bottom panel shows the negative BOLD signal areas for passive touch to the palm and arm; these regions overlapped greatly. The brain map in the bottom panel is to neurological convention (left is left).

Differences between active and passive touch were mainly seen in the left sensorimotor cortex, as would be expected for the right, contralateral, and limb movement. During active stroking of the palm and arm, a network of left cortical areas showed significant positive BOLD signals including: SI, MI, premotor cortex, and somatosensory association areas (BA05 and BA07; see Table 1). These activations likely reflect the interaction of motor and sensory components during active, self-touch including movement planning, co-ordination and sensory feedback from the skin of the right palm. Furthermore, in contrast to the positive BOLD signals during active touch compared to the resting baseline (see Figure 1, top left panel and Figure 3 top panel), a significant negative BOLD signal was found in the left SI during passive touch to both the palm and arm (Figure 3). This covered an extensive part of SI and also spread into the left MI. A further difference was seen in the insula cortices: there were significant bilateral positive BOLD signals during passive touch to both the palm and arm, compared to the resting baseline. In contrast, during active touch, only the right insula showed a significant BOLD signal in self-palm stroking, compared to the resting baseline.

## Discussion

The present study used light brush stroking to elicit cortical responses from glabrous and hairy skin. The BOLD signal was modulated by the skin site (palm or arm) and by the type of stroking (active or passive), and an interaction was seen in the right SI (contralateral to the touch) in BA03 between active and passive stroking of the palm and the arm. Here, a clear somatotopical difference was observed between the representations of each body site, the palm being a much larger representation and the arm was represented more laterally, and in both respective areas, active stroking gave an increased BOLD signal over passive stroking. Previous studies have also found that the arm is represented more laterally than from the Penfield and Rasmussen (1950) cortical homunculus, e.g., Olausson et al. (2002) and Gordon et al. (2011). The larger representation of the glabrous skin of the hand would be expected in SI, due to both the increased peripheral receptor density and differences in the type of receptors present (i.e., the glabrous skin also sends RAI afferents to the cortex), however, the finding of active touch, in general, producing significantly higher BOLD signals was more controversial. The present study adds to the evidence that the signal in contralateral SI is indeed modulated, for both glabrous and hairy skin sites, although it is likely that the afferent signal to SI touch may be modulated in a context-dependent manner (Chapman and Ageranioti-Bélanger, 1991; Ageranioti-Bélanger and Chapman, 1992; Chapman, 1994; Jackson et al., 2011). If there is behavioral relevance for the tactile information gained from active touch, the cancellation effect from the sensory prediction of the consequences of the motor efference may be countermanded by an internal mechanism to attend to the touch afference. This attentional or cognitive internal drive may determine whether the signal to contralateral SI is attenuated or amplified. It is therefore likely that the input to SI is nevertheless subject to movement-related gating, which would decrease the incoming signal during active touch; however, the efference copy of the movement and/or the prediction of its sensory consequence may be countermanded by the demands of the task.

The extent to which the signal is attenuated or amplified may be modulated by factors such as attention and motor strategy, depending on the situation (Chapman, 1994). Previous studies have shown that there is not necessarily a direct relationship between activity in afferent touch systems and changes in the BOLD signal and the relationship can vary with attention (Johansen-Berg and Lloyd, 2000; Arthurs et al., 2004). During active touch, interactions between motor and sensory cortices may also regulate context-dependent information processing in SI (Lee et al., 2008). Evidence from humans for the modulation of this movement-related gating comes from Master and Tremblay (2009, 2010), who found that active tactile exploration increases corticomotor excitability when tactile information is sought, rather than ignored. In present study, the participants may have paid more attention to the active stroking as they did not have visual feedback from their touch, thus giving the higher signal in SI. Furthermore, the current study provides evidence that this signal was only modulated in BA03; no significant modulations in the level of the positive BOLD signal were seen between active and passive touch for other areas during stroking of the palm or arm. There was an interaction between the touch conditions in SII: all the conditions apart from active touch to the palm elicited significant positive BOLD signals bilaterally in SII. Active touch to the palm, however, only showed significant positive BOLD signal changes in the right SII. The present results suggest that somatosensory signals arriving in SII from both palms may culminate in a gating effect to focus on the most relevant input for the current task. Different patterns of activity in SII may aid in attention to a certain body area, especially during bilateral body interactions with input from the same body area on both sides.

We investigated self-touch with an instrument, rather than the direct skin-to-skin contact. It was deemed that a brush was a better, more controlled stimulus for factors such as temperature and social interactions. Furthermore, the participants were able to train in stroking with the brush, which also allowed a constant force to be applied between the conditions. A potential confound of the finding of the active/passive touch modulation in BA03 may have been small differences in the applied force of the brushing. An increased signal in SI for both the palm and arm active brushing may have been due to the participant stroking themselves with more force. However, the force from the brush would not have been too different as the hairs on the brush provided only light forces (typically <1 N) and it was difficult to achieve a heavy force with the brush, unless it was pushed into the skin. As the participant was able to practice stroking with the brush beforehand, the experimenter was able to make sure that the brushing was consistent within and between participants. Although, the task was repetitive, the participants were required to pay attention to the task at hand and no overall decrease in the modulation of the BOLD signal was seen over the experiments. Also, as the effect was only found in BA03, this points to an attentional mechanism for gating of the initial processing of incoming touch information. It is likely that self-touch using skin-to-skin contact would produce a somewhat different signal. With this reasoning, the left sensorimotor touch network found during active touch in the present study may be different when touch is directed to another object that is not the self.

During active touch, a network of sensorimotor areas was recruited in the left cortex, contralateral to the moving limb. Strong, positive BOLD signals were observed particularly in left SI and MI, with other sensory (somatosensory association cortex) and motor (premotor cortex) areas also following the same pattern of modulation during active touch. A similar network of connectivity between motor, premotor, sensory, and sensory association areas has been demonstrated in the monkey (Morecraft et al., 2004, 2012). In the present study, a negative BOLD signal was found in the left sensorimotor cortex during passive touch. There are a number of potential explanations for this including “vascular blood stealing” from surrounding areas. This entails that an area showing a positive BOLD signal can produce a nearby negative BOLD signal due to blood being diverted from a nearby inactive area. In the present study, this is unlikely as there was no positive BOLD signal in close proximity to the negative BOLD signal during the passive stroking. Another potential explanation may be that the negative BOLD signal may have been due to a “memory” of previous active touch modulations, such as, residual neuronal inhibition or a compensatory decrease in blood flow after the large BOLD signal from active touch. However, the BOLD signals returned to the resting baseline level after each touch stimulus had ceased and the stimuli were randomized, making this explanation less likely. Negative BOLD signals have been shown to correlate with decreases in neuronal activity (Shmuel et al., 2006), which could manifest as a change in ongoing brain rhythms. We believe that the negative BOLD signal in the ipsilateral, left SI during passive touch was due to a change in the neuronal activity as a result of unilateral touch to the left side of the body. Recent papers have shown that somatosensory stimulation of one hand elicits positive BOLD signals in the contralateral cortex with accompanying negative BOLD signal modulations in the ipsilateral cortex (Hlushchuk and Hari, 2006; Kastrup et al., 2008; Klingner et al., 2010, 2011; Schäfer et al., 2012).

EEG studies have shown that a unilateral somatosensory stimulus to one hand will elicit an event-related potential in the contralateral SI, which is accompanied by a concurrent event-related desynchronization of the ongoing mu rhythm over both somatosensory cortices, although, more strongly on the contralateral side to the somatosensory event (Korvenoja et al., 1995; Nikouline et al., 2000). The evidence shows, that a somatosensory event to one side of the body only, will elicit a response in the contralateral SI, which has an effect on the ipsilateral SI, via transcallosal connections between the sensorimotor cortices (Fabri et al., 1999; Ragert et al., 2011). We postulate that cross-talk between sensorimotor cortices will facilitate attention and differentiation of somatosensory information from each side of the body. Specifically, the inhibitory signal that is sent from the activated SI to the opposite SI may aid in bimanual processing and the interpretation of tactile localizations and interactions (Kastrup et al., 2008). The differences in the BOLD signals may play a role in distinguishing between active, self-touch (with the internally-generated efferent copy feedback) from passive, external touch (with no predictions of the consequences). It is possible that the shift between significant positive and negative BOLD signals to active and passive stroking, respectively, do also reflect the timing and expectancy of previous and subsequent touch stimuli during the paradigm. Also, only during passive touch was bilateral insula activity seen. This again may be part of a network of sensory areas that help distinguish both between self- and other touch.

In conclusion, the present study found a distinct cortical pattern associated with each of the four touch conditions. Differences between touch to the palm and arm were found: the glabrous skin of the palm showed a significant representation in the contralateral, right SI, whereas this signal was less extensive for touch to the arm, which relates to the mechanoreceptive input and usage for discriminative touch. The significant, positive BOLD signal was modulated in the respective body site areas in BA03, where active touch gave an increased signal over passive touch. Active touch using the right hand/arm elicited a network of positive BOLD signal changes in left sensorimotor areas; conversely, a negative BOLD signal for passive touch in the left SI. The present study has implications for understanding how touch information is processed and gated according to the behavioral situation and could be used to refine touch interactions with everyday objects in rehabilitation studies. Furthermore, touch processing appears to be heavily influenced by the reasons and attentional demand of the task at hand, where both motor and sensory information is relevant. Future studies will explore factors, such as, attentional modulations to touch, including bilateral touch and touch at other body sites, and how active movement influences touch both to the self and other objects.

### Conflict of interest statement

The authors declare that the research was conducted in the absence of any commercial or financial relationships that could be construed as a potential conflict of interest.
